# Wear Behaviour of Polymer-Infiltrated Network Ceramics, Lithium Disilicate and Cubic Zirconia against Enamel in a Bruxism-Simulated Scenario

**DOI:** 10.3390/biomedicines10071682

**Published:** 2022-07-12

**Authors:** Andrea Baldi, Massimo Carossa, Allegra Comba, Mario Alovisi, Felice Femiano, Damiano Pasqualini, Elio Berutti, Nicola Scotti

**Affiliations:** 1 Department of Surgical Sciences, CIR Dental School, University of Turin, Via Nizza 230, 10126 Turin, Italy; andrea.baldi@unito.it (A.B.); allegra.comba@unito.it (A.C.); mario.alovisi@unito.it (M.A.); damiano.pasqualini@unito.it (D.P.); elio.berutti@unito.it (E.B.); nicola.scotti@unito.it (N.S.); 2 Restorative Dentistry, Multidisciplinary Department of Medical-Surgical and Dental Specialties, University of Study of Campania, “Luigi Vanvitelli”, Via De Crecchio 6, 83138 Naples, Italy; femiano@libero.it

**Keywords:** polymer-infiltrated network ceramics, zirconia, wear, bruxism

## Abstract

The present study aimed to evaluate the wear rate of polymer-infiltrated network composites and ceramics against enamel in a bruxism-simulated scenario. Ninety-six (*n* = 96) molars were divided into six groups (*n* = 16) according to their occlusal material: group 1—a polymer-infiltrated network ceramic (PINC); group 2—a second polymer-infiltrated network ceramic (PINC2); group 3—nanohybrid resin-based composite (CO); group 4—cubic zirconia (ZR); group 5—lithium disilicate (LS); and group 6—sound enamel (EN). A laser scanner was used to digitalize all of the occlusal surfaces before and after a fatigue test, which was conducted with a chewing simulator set at 80 *N* and semicircular movement in order to simulate bruxist movement and loads. Statistical analysis of volume loss was performed with a one-way ANOVA and post hoc Bonferroni test. ZR had significantly inferior wear to PINC (*p* ≤ 0.01) and CO (*p* = 0.04). LS wore the antagonist enamel significantly more than PINC, CO, PINC2 and EN (*p* ≤ 0.01). On the other hand, ZR wore the antagonist enamel significantly more than CO (*p* ≤ 0.01) and PINC2 (*p* = 0.05). In conclusion, PINCs better preserved antagonist enamel at the expense of a higher wear of their own. LS causes significantly higher enamel wear compared with PINCs. ZR caused significantly higher enamel wear compared with CO and PINC2, but it was wear-resistant.

## 1. Introduction

In recent years, the attention of the scientific community to the occlusal wear problem has increased as a consequence of the evidence showing a high prevalence of bruxism and the introduction of new restorative materials that have been applied in worn dentition rehabilitations and might have an influence on antagonist wear [1,2,3].

Bruxism is defined as a diurnal or nocturnal parafunctional activity characterized by the clenching or grinding of the teeth and/or by the bracing or thrusting of the mandible [4]. During physiological masticatory function, humans develop forces that easily reach 200 *N*, depending on a variety of factors [5,6]. In bruxism patients, these forces can be up to six times more intense [6], with an abnormal increase in the frequency and duration of interdental contacts, which significantly contribute to tooth wear [7]. As reported in the literature since the 1990s, in a physiological scenario, wear still happens, resulting in a slow and progressive flattening of cusp tips on the posterior teeth and incisal edges on the anterior teeth [8]. It has been estimated in vivo that the physiological enamel wear in molars can range around 29 µm per year [9]. However, when bruxism or other pathological conditions are involved, teeth are worn significantly more in the same amount of time, progressively leading to a lack of aesthetic and function, loss of vertical dimension and possible consequences on the temporo-mandibular joint. With the demographic change of the population, the high prevalence of bruxism and the high aesthetic demands of modern society, it is, therefore, not surprising that modern restorative dentistry frequently has to deal with severely worn dentitions.

In order to re-establish a proper function and aesthetic with minimal invasiveness, several new materials have been proposed with the introduction of CAD/CAM technology, including polymer-infiltrated network ceramics (PINC), hybrid ceramics, lithium disilicate and zirconia, for permanent restorations [10]. The aim of these newly developed materials is to offer a biomechanical behaviour similar to that of enamel, ensuring aesthetic, function and vertical dimension stability. In order to achieve this, these materials should ideally be wear-resistant, but should also preserve the antagonist enamel. Since their introduction, several studies have investigated the wear characteristics of these materials over time, as well as the wear that these materials cause on the antagonist tooth. Ludovichetti et al. [11] tested five different materials for CAD/CAM workflow, plus bovine enamel, in a simulated chewing scenario. The authors’ findings highlighted how PINCs wore the antagonist less when compared with zirconia and glass–ceramics. Consistencies were reported by Habit et al. [12], who found higher wear of the enamel when matched with zirconia. In contrast, different Authors [13,14,15,16] found different results, showing how zirconia produced less antagonist wear in comparison with PINC materials. These inconsistencies within apparently similar in vitro studies may be related to the different testing procedures adopted, as well as to the different polishing treatments of each material. However, research on the mechanical properties of PINCs and ceramics remains open because of a strong and fast evolution in their composition.

As a matter of fact, the enamel wear pattern is unique due to its micro- and nanostructure and its unique anisotropic behaviour [17]. According to Jing Xia et al. [18], enamel shows different responses to the increasing magnitude of loads: plucking (nanosphere loss when the strength of the bonding protein ‘glue’ is exceeded), plastic deformation (compression to gradually bend nanofibres and squeeze the protein layer) and fragmentation (nanofibres fracture when the strength of the H-bonds that bind smaller nanoparticles into nanospheres is exceeded). In their study, Jing Xia et al. also reported that a lower contact pressure is required to generate a certain response with shear forces applied perpendicular to the long axes of crystallites, rather than axial forces parallel to them. Bruxist patients might, therefore, have peculiar enamel wear behaviour due to the stronger forces involved and higher shear stresses generated by the grinding parafunctional movements.

That being said, even if several studies have evaluated the wear performance of enamel and materials processed through CAD/CAM technologies, there is little knowledge about their behaviour when subjected to a bruxism-simulated load and pattern. Therefore, the aim of the present study was to evaluate, through superimposition analysis, the wear behaviour of PINCs, lithium disilicate and cubic zirconia against enamel in a bruxism-simulated scenario. The null hypotheses were that the tested materials (1) had a similar wear behaviour to each other and (2) induced the same amount of wear to the antagonist enamel.

## 2. Materials and Methods

### 2.1. Sample Selection

Ninety-six (*n* = 96) human second-lower molars and ninety-six second-upper molars with mature apices, extracted within three months from the test for periodontal reasons, were selected. The study was granted ethical approval by the local ethics committee of the Dental School, University of Turin (DS-2018_No.001). The inclusion criteria were as follow: no evident occlusal abrasions, sound enamel in the cusps area, no caries, similar crown size (10 mm ± 2 mesio-distal, 10 mm ± 2 bucco-oral) and morphology, and no fracture, cracking or demineralization under transillumination with 6× optical magnification. Ultrasonic scaling and polishing were performed for surface debridement. After that, the samples underwent disinfection (0.5% chloramine for 48 h) and were then stored in distilled water at 37 °C.

### 2.2. Sample Preparation

**Figure 1:**
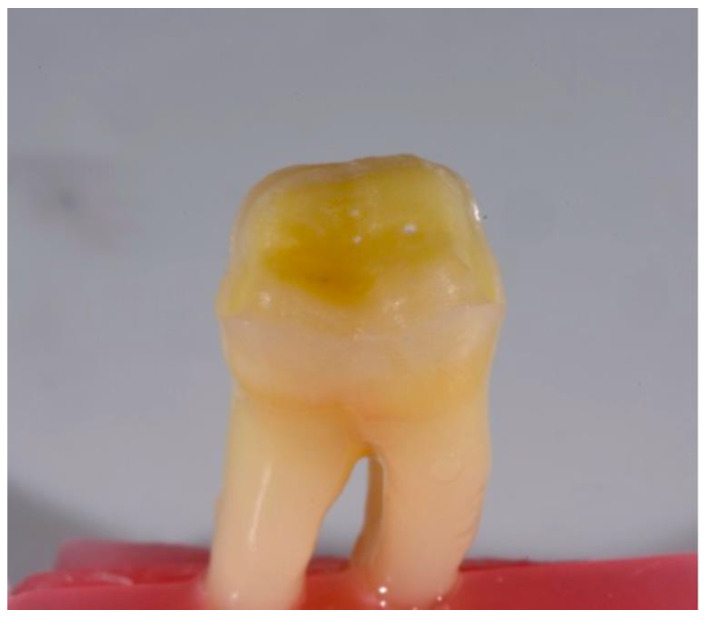
An example of a prepared lower molar specimen. Standardized occlusal reduction of 1.5 mm was performed following occlusal anatomy; buccal and oral finish lines with a 2 mm-long bevel were created all around the tooth.

The lower molars underwent a standardized full bevel tabletop preparation in order to mimic a worn dentition case in which modern PINCs, lithium disilicate and cubic zirconia could be applied to re-establish occlusal anatomy. Preparations were performed by the same expert operator (more than 10 years of practice in restorative dentistry) with a standardized occlusal reduction of 1.5 mm with a cylindrical bur (6836.KR.014, Komet, Schaumburg, IL, USA) following occlusal anatomy. Buccal and oral finish lines were created with a 45° inclined long chamfer bur (8862.314.012, Komet, Schaumburg, IL, USA) in order to create a 2 mm-long bevel all around the tooth (Figure 1). This preparation was meant to be minimally invasive, while also increasing the exposed enamel surface and removing non-sustained enamel [19].

Lower molars were then divided into 6 groups (*n* = 16) according to their occlusal material: group 1 (PINC)—a PINC (GrandioBlocks, Voco, Cuxhaven, Germany); group 2 (PINC2)—a second PINC (Cerasmart, GC, Tokyo, Japan); group 3 (CO)—nanohybrid resin-based composite (RBC) (Venus Pearl, Kulzer, Hanau, Germany); group 4 (ZR)—cubic zirconia (Katana STML, Kuraray Noritake, Tokyo, Japan); group 5 (LS)—CAD/CAM lithium disilicate (E-Max CAD, Ivoclar Vivadent, Shaan, Lichtenstein); group 6 (EN)—control group with sound enamel. A general description of the materials used in the present study and their manufacturers and composition are listed in Table 1.

Lower restored molars (except for group 6) and upper sound molars were randomly paired and fixed in their proper anatomical position with silicone putty inside a gypsum model mounted in an articulator. The positioning was carried out in order to maintain a proper cusp–pit ratio.

**Figure 2:**
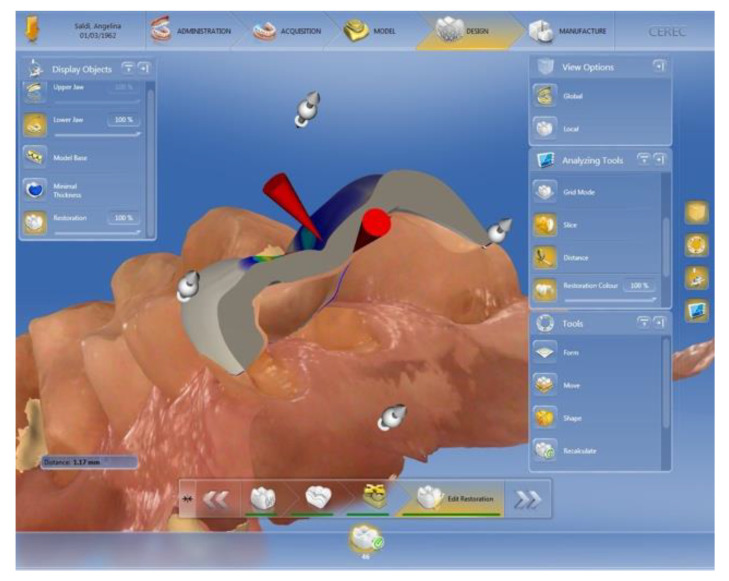
CAD design of tabletop restorations, controlling standardized anatomy and thickness.

Prepared teeth, except for those in group 3, were scanned with an intraoral camera (Cerec Omnicam AC, Dentisply, Sirona, Konstanz, Germany) and the restorations were designed with CAD software (Cerec 4.5.2, Dentisply, Sirona, Konstanz, Germany). Particular attention was given to occlusal modelling in order to have precisely 3 mm^2^ of contact area between the opposing teeth, distributed in tripod contact. Moreover, in order to minimize the variability among samples, the cusp inclination was set to a default of 30° and secondary crests were removed (Figure 2).

Finally, the tabletops were milled with a CAM system in the extra-fine mode, following the manufacturer’s parameters for the respective materials (Cerec MC XL, Dentsply, Sirona, Konstanz, Germany). Once milled, LS and ZR were crystallized with their dedicated protocols (Cerec Speedfire, Dentsply, Sirona, Konstanz, Germany). CO overlays (group 3) were modelled by an expert technician with an oblique layering technique. Both CO and EN were checked with the CAD software to set a proper contact area with the antagonist through selective grinding.

At this point, since it has been widely demonstrated that surface roughness is a crucial factor that influences wear, in particular when horizontal movements are emphasized, all samples were progressively polished with dedicated diamond burs and rubber points [8]. For the same reason, no glazing was applied to the ceramic materials, which were only mechanically polished. Surface porosity, another factor that might influence wear behaviour, can be considered negligible in this protocol due to the sintering procedures followed, which have been widely tested in the literature.

Each overlay was then luted following the manufacturer’s instructions, which are summarized in Table 2.

An optical laser scanner (LAS-20; SD Mechatronik GmbH, Westerham, Germany) with a horizontal resolution of 10 µm and a theoretical vertical resolution of 0.8 µm was used to digitalize all of the occlusal surfaces, both those of the lower and upper molars, before the fatigue test. All files were exported in STL format for further analysis.

### 2.3. Fatigue Test

Both upper and lower paired samples were positioned in a metal holder and embedded with acrylic resin 1 mm below the CEJ junction. The fatigue test was conducted using a chewing simulation machine (CS-4.4 professional line, SD Mechatronik, Westerham, Germany) that allowed testing vertical and horizontal movements simultaneously under thermodynamic conditions. The upper molars were fixed in the upper holder, while the lower molars were fixed in the lower holder. Specimens were moved on the two axes in order to respect the CAD project, with the palatal cusps of the upper molars occluding with the central fossa of the lower molars.

In order to simulate a bruxism scenario, a cyclic fatigue test was performed for 500,000 cycles with a load of 8 kg on each tooth (about 79 Newtons of chewing force), a semicircular movement (circle diameter: 6 mm; maximum intrusion depth: 3 mm; and speed: 4 mm/s) and a frequency of 1.2 Hz. During the chewing process, 3000 thermocycles were applied with water medium between temperatures of 5 °C and 55 °C and a dwell time of 60 s [20].

### 2.4. Quantitative Analysis of Wear

After the fatigue test, each sample was submitted to a second scan with the same baseline parameters and scanner in order to achieve consistency between the data. The volumetric wear of each specimen was calculated using dedicated software (Geomagic Control Software, 3D Systems, Darmstadt, Germany), superimposing the baseline and T1 data points of the whole occlusal surface and evaluating the volume loss, expressed in mm^3^.

Some random specimens were then prepared with 4% glutaraldehyde for 12 h at 4 °C. After the preparation process, deionized water was used to rinse the specimens and they were initially dehydrated with an increasing concentration of ethanol (25%, 50%, 75%, 95% and 100%). The specimens were then dried using a Critical Point Dryer (Leica EM CPD300, Germany) and were sputter-coated with gold (JFC 1600, JEOL, Tokyo, Japan), and finally observed under SEM at 10 kV voltage. A series of micro-photographs were acquired at ×5000 and ×10,000 magnifications in order to view the surface morphology.

### 2.5. Statistical Analysis

Data concerning volumetric wear were analysed with the Kolmogorov–Smirnov test for normality and revealed a normal distribution. They were, therefore, analysed with a one-way ANOVA test and post hoc Bonferroni test. A *p*-value of *p* < 0.05 was considered to indicate statistical significance. All statistical analyses were performed using the STATA software package (ver. 12.0; StataCorp, College Station, TX, USA).

## 3. Results

The mean volumetric wear for each group ± standard deviation, expressed in mm^3^, is reported in Table 3. Both the volumetric loss of the tested materials (lower molar) and the respective enamel wear they caused (upper molar) were taken into account.

The ANOVA test reported significant differences (*p* ≤ 0.01) between the tested materials’ volume loss. Subsequent post hoc test revealed that ZR had a significantly inferior wear compared to PINC (*p* ≤ 0.01) and CO (*p* = 0.04).

**Figure 3:**
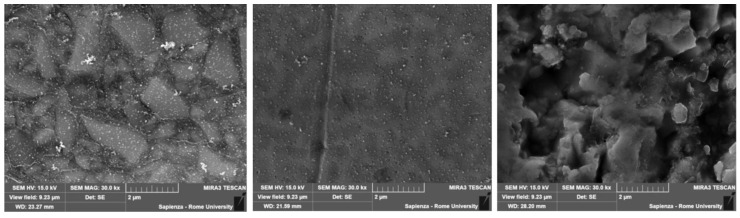
SEM analysis showed different surface characteristics of the tested materials: PINC (on the left) showed uniform wear with a low visual roughness, even if an initial enucleation of some filler particles and some signs of subsuperfical cracks could be observed. LS (in the centre) showed mineral particles on the surface, which could enhance the roughness and, therefore, the abrasive effect towards the antagonist enamel. The cubic zirconia (on the right) showed a superficial delamination, which made this material less rough and, consequently, less aggressive towards the enamel.

ANOVA test also reported significant differences (*p* ≤ 0.01) between antagonist wear. The subsequent post hoc test revealed that LS wore the antagonist enamel significantly more than PINC, CO, PINC2 and EN (*p* ≤ 0.01). On the other hand, ZR wore the antagonist enamel significantly more than CO (*p* ≤ 0.01) and PINC2 (*p* = 0.05). Representative SEM images showed the wear pattern of the tested materials (Figure 3).

## 4. Discussion

As a consequence of increasing bruxism, enamel wear has been widely discussed in order to perform minimally invasive occlusal rehabilitations. Even if a lot of modern materials claim to be “enamel-friendly” and try to emulate enamel mechanical properties, there are still progressions that have to be made. This is due to the fact that enamel has a unique mechanical behaviour connected to its crystal arrangement and stabilizing proteins [21,22] that led to defining it as “metallic-like” [23]. The present study aimed to evaluate, in a bruxism-simulated scenario (high forces, high number of cycles with lateral movements), the wear behaviour of PINCs, lithium disilicate and cubic zirconia against enamel.

Based on the results obtained in the present study, the tested null hypotheses were rejected: statistically significant differences were found between the wear behaviour of different materials and the wear they induced to the enamel.

Concerning the quantitative loss of volume of enamel against different materials, the obtained results were partially in accordance with Habib et al. [12]. In their study, nanohybrid RBCs caused the least wear to enamel, which was also observed in the present study. Moreover, they reported that the two more aggressive materials against natural enamel were zirconia and lithium disilicate. However, the zirconia tested by Habib et al. was multi-layered, with a high amount of cubic phase, which is more prone to mechanical degradation and might lead to less uniform prism exposure and a consequent superficial roughness increase, which, therefore, explains the higher wear induced by this material compared with that obtained in the present study [24]. This was also confirmed by Sripetchdanond et al. [13], who concluded that the materials inducing the least wear to enamel were RBC and zirconia, as well as different reviews that showed how zirconia has friendly wear-behaviour against enamel [14,15,16]. The present results were also confirmed by Ludovichetti et al. [11], who reported a high abrasiveness of lithium disilicate, while nanofilled RBC and PINC were more antagonist-friendly. However, it should be noted that Ludovichetti et al.’s study obtained higher wear values of the enamel caused by LS and ZR wear values close to those of the LS. This may suggest that the behaviour of ZR and LS might change when subjected to increased chewing loads and number of cycles, as was the case in the present study. Another possible explanation is the polishing procedure that they performed, which was different from that in the present study. As shown by several papers, in order to reduce the extent of wear of the antagonist enamel, the polishing procedure is fundamental, even if ZR seems enamel friendly even after simulated clinical adjustments [25]. Accordingly, in several other studies on composites, all resin-based materials showed minimal wear of the antagonist enamel at the expense of higher wear of their own [26,27]. This is probably related to the mechanical properties of these materials, which have an inferior fracture toughness compared with enamel and, therefore, undergo fatigue failure prior to enamel itself [8]. Estimating a precise fracture toughness of enamel in vitro has proven difficult; however, most of the literature data are superior to those of tested PINCs [28]. This is also in accordance with the present study’s result of CO showing slightly inferior enamel wear compared with PINC and PINC2, which possess superior fracture toughness [29]. Despite everything, it can be considered that the difficult standardization of the enamel roughness of the antagonist samples, associated with the surface roughness of the tested materials, which has not been measured, may represent limitations of the present study that will be considered in future research.

Considering the tribological properties of the tested materials, it is well known that, during fatigue tests, PINC, CO and PINC2 show crack propagation around the fillers, with a superior frequency in the CO and PINC groups. This phenomenon, described in the literature as subsurface crack propagation, causes filler enucleation and detachment [30]. As a consequence, the surface roughness increases, negatively affecting the abrasiveness of the PINCs towards the antagonist enamel [31,32]. On the other hand, ceramics such as LS and ZR do not possess plastic behaviour: under fatigue, cracks probably propagate within the structure instead at the surface level. Therefore, it is not surprising that their wear behaviour mostly consisted of delamination starting from wear facets [33]. Previous studies showed how the ZR delamination phenomenon seems to be more similar to the sound enamel wear mechanism, with several cracks regularly distributed throughout the entire extension of the wear pattern [34,35]. On the contrary, the LS group showed a more irregular surface and crack propagation pattern, both inside and outside of the wear scar’s area [36].

Finally, with regards to the tested materials’ wear, unsurprisingly, ZR showed significantly better performance compared with PINC and CO. Even if not significant, ZR also had inferior average wear compared with LS, enamel and PINC2. This is surely related to the higher fracture toughness and hardness of zirconia, which is also less susceptible to crack growth and fatigue compared with LS [37]. Speaking of resin-based materials, even from a clinical point of view, there is evidence that PINC crowns preserve occlusal anatomy only in 26.5% of cases versus 96% of ceramic crowns after 3 years [38]. This was also confirmed by Mormann et al., who demonstrated how PINCs are more susceptible to volumetric wear, but more enamel-friendly compared with most ceramic materials, in accordance with the present study’s results [39].

## 5. Conclusions

Within the limitations of the present in vitro study and based on the obtained results, it can be concluded that:

PINCs tend to preserve antagonist enamel, at the expense of a higher wear of their own.

LS causes significantly higher enamel wear compared with all PINCs.

ZR causes significantly higher enamel wear compared with CO and PINC2, but it is wear-resistant.

Further studies are necessary to improve the knowledge of how these materials behave against natural enamel, especially when higher loads and frequencies are applied.

## Figures and Tables

**Table 1:** General description of the main materials used in the present study, along with their commercial name, manufacturer and composition.

General Description	Commercial Name	Manufacturer	Composition
Polymer-infiltrated network ceramic (PINC)	Grandio Blocks	VOCO	86 wt% nanohybrid filler in a polymeric matrix UDMA + DMA
Polymer-infiltrated network ceramic (PINC2)	Cerasmart	GC	71 wt% silica and barium glass nanoparticles, Bis-MEPP, UDMA, DMA
Direct nano-hybrid resin-based composite (CO)	Venus Pearl	Kultzer	59 wt% TCD-DI-HEA, 58% UDMA, 2% barium, 1% fluoride aluminium
Cubic ZrO_2_ (ZR)	Katana STML Zirconia	Kurakay	88 wt% zinc oxide, 9% yttrium oxide, 3% hafnium dioxide, 0.5% aluminium oxide
CAD/CAM lithium disilicate (LS)	E-max CAD	Ivoclar	Silicon dioxide, lithium oxide, potassium oxide, phosphorus pentoxide, zinc oxide

**Table 2:** Detailed adhesive procedures performed on the substrate and the various tested materials.

Substrate	Adhesive Procedure Performed
Tooth	Enamel etching for 30 s, dentin etching for 15 s with 37.5% phosphoric acid (K-etchant, Kuraray Noritake, Tokyo, Japan), 30 s rinsing, 30 s air-drying, primer application (Optibond FL Primer, Ker, Orange, CA, USA) over the surfaces with a light scrubbing motion 15 s, then gentle air drying and bonding application (Optibond FL Bond, Kerr, Orange, CA, USA)
PINC	Sandblasting with aluminium oxide (50 μm) at 1.5–2 bar, cleaning with an ultrasonic bath, heated silane 60 s (Ceramic Primer PLUS, Kuraray Noritake, Tokyo, Japan), drying 10 s with air and applying bonding (Optibond FL Bond, Kerr, Orange, CA, USA). A flowable RBC was then applied (Clearfil Majesty ES, Kuraray Noritake, Tokyo, Japan) and light-cured for 2 min at 1000 mW/cm^2^ (Cefalux 2, Voco, Cuxhaven, Germany)
PINC2	Hydrofluoric acid at 5% (IPS Ceramic etching gel, Ivoclar, Shaan, Lichtenstein) 60 s, rinsing 60 s, cleaning with an ultrasonic bath, drying, applying silane, bonding and flowable RBC as described for PINC
CO	Same procedure as that performed for PINC
ZR	Sandblasting with aluminium oxide (25 µm) at 1.5–2 bar, cleaning with ultrasonic bath, primer application (Ceramic Primer PLUS, Kuraray Noritake, Tokyo, Japan), drying 20 s. Bonding and flowable RBC as described for PINC
LS	Hydrofluoric acid at 5% (IPS Ceramic etching gel, Ivoclar, Shaan, Lichtenstein) 30 s, followed by the same procedure as that performed for PINC2

**Table 3:** Mean ± standard deviation, expressed in mm^3^, for both tested materials and their antagonists.

	Tested Material Wear (Lower Molar Wear)	Antagonist Wear (Upper Molar Wear)
Polymer-infiltrated network ceramic (PINC)	0.0259 ± 0.008	0.0171 ± 0.005
Second polymer-infiltrated network ceramic (PINC2)	0.0154 ± 0.008	0.0128 ± 0.007
Nano-hybrid resin-based composite (CO)	0.0216 ± 0.006	0.009 ± 0.004
Cubic zirconia (ZR)	0.0098 ± 0.004	0.0314 ± 0.008
Lithium disilicate (LS)	0.0204 ± 0.007	0.0439 ± 0.009
Enamel (EN)	0.0205 ± 0.006	0.0211 ± 0.007

## Data Availability

The datasets generated during and/or analysed during the current study are available from the corresponding author on reasonable request.
